# Supplementary material for: Predicting the Association of Metabolites with Both Pathway Categories and Individual Pathways
Source: Metabolites. 2024 Sep 21;14(9):510. doi: 10.3390/metabo14090510 (PMC11433779; doi:10.3390/metabo14090510)
Supplement: Supplementary file 1 [file metabolites-14-00510-s001.zip › metabolites-3176207-supplementary.pdf]

Table S**1.** Optimal hyperparameters by dataset.

Dataset	Scaled	Activation Function	Normalization Function	Loss Function	Hidden Layer Size	# Layers	Dropout	Learning Rate	Beta 1	Beta 2	EPS	Weight Decay	Classification Threshold
**Level 3**	TRUE	ReLU	BatchNorm	bce	1706	4	0.0735	2.0307E-06	0.2193	0.8135	0.000144	0.036613	0.5791
	FALSE	PReLU	BatchNorm	bce	2637	4	0.0322	2.7362E-05	0.2606	0.3226	9.433758E-05	0.034935	0.4048
**Combined**	TRUE	CELU	BatchNorm	bce	3518	3	0.0845	9.9880E-05	0.1462	0.7005	0.000305	0.000157	0.6059
	FALSE	PReLU	BatchNorm	bce	3467	3	0.0654	0.00061	0.0324	0.1733	0.000451	2.604479E-05	0.4078

Table S2. Model performance metrics for each dataset.

Dataset	Scaled	Metric	Mean Score	Standard deviation
**Combined**	True	Accuracy	0.9930	0.0011
		F1 score	0.8013	0.0223
		MCC	0.7997	0.0209
		Precision	0.7551	0.0396
		Recall	0.8551	0.0129
	False	Accuracy	0.9926	0.0003
		F1 score	0.7743	0.0085
		MCC	0.7706	0.0086
		Precision	0.7735	0.0176
		Recall	0.7754	0.0130
**Level 2**	True	Accuracy	0.9473	0.0082
		F1 score	0.7556	0.0262
		MCC	0.7275	0.0294
		Precision	0.7528	0.0575
		Recall	0.7629	0.0340
	* False	Accuracy	0.9592	0.0027
		F1 score	0.8069	0.0114
		MCC	0.7844	0.0129
		Precision	0.8128	0.0224
		Recall	0.8019	0.0193
**Level 3**	True	Accuracy	0.9930	0.0015
		F1 score	0.6529	0.0359
		MCC	0.6547	0.0314
		Precision	0.6830	0.0883
		Recall	0.6440	0.0821
	False	Accuracy	0.9902	0.0041
		F1 score	0.6101	0.0628
		MCC	0.6182	0.0481
		Precision	0.5593	0.1238
		Recall	0.7129	0.0787

** The results highlighted in yellow were provided by the work of Huckvale and Moseley in the supplemental materials of the manuscript of DOI: https://doi.org/10.3390/metabo14050266*

Table S3. Sum of True/False Positives/Negative metrics across CV iterations by pathway hierarchy level (L2 or L3). Results are from training on the combined and scaled dataset.

Dataset	Pathway Hierarchy Level	Scaled	Metric	Sum	Count (number of pathway categories times 100 CV iterations)
**Combined**	2	TRUE	True positives	69,342	1,200
			True negatives	597,569	1,200
			False positives	12,142	1,200
			False negatives	2,963	1,200
		FALSE	True positives	63,596	1,200
			True negatives	599,093	1,200
			False positives	10,963	1,200

			False negatives	8,677	1,200
	3	TRUE	True positives	77,484	17,200
			True negatives	9,639,265	17,200
			False positives	36,124	17,200
			False negatives	21,911	17,200
		FALSE	True positives	69,546	17,200
			True negatives	9,646,907	17,200
			False positives	28,137	17,200
			False negatives	29,881	17,200

Table S4. Characteristics and performance metrics for each L2 and L3 pathway. Results derive from training on the combined and scaled dataset.

Pathway ID	Number of metabolites	Number of non-hydrogen atoms	MCC score	True positives	True negatives	False positives	False negatives
**Amino acid metabolism**	611	10774	0.8409598893071340	5792.0	49225.0	1555.0	391.0
**Biosynthesis of other secondary metabolites**	1486	38836	0.9081988788786460	14491.0	40591.0	1735.0	382.0
**Carbohydrate metabolism**	514	11485	0.8902161978132910	4837.0	50831.0	898.0	195.0
**Chemical structure transformation maps**	437	9298	0.7442351678288250	3885.0	50243.0	1999.0	483.0
**Energy metabolism**	173	4470	0.8441864642903980	1539.0	54626.0	398.0	157.0
**Glycan biosynthesis and metabolism**	324	18936	0.8687650948335800	3133.0	52902.0	730.0	172.0
**Lipid metabolism**	677	22159	0.9480287144475790	6788.0	49618.0	535.0	119.0
**Metabolism of cofactors and vitamins**	549	15569	0.8192698358391200	5099.0	49542.0	1800.0	269.0
**Metabolism of other amino acids**	274	4989	0.8341470754511110	2554.0	53293.0	760.0	217.0
**Metabolism of terpenoids and polyketides**	1093	34676	0.9402708096069460	10590.0	44929.0	756.0	317.0
**Nucleotide metabolism**	169	3592	0.9420564036585730	1650.0	55260.0	166.0	33.0
**Xenobiotics biodegradation and metabolism**	939	16780	0.9349955392946330	8984.0	46509.0	810.0	228.0
**00010 Glycolysis / Gluconeogenesis**	43	997	0.7521514556232700	349.0	55995.0	129.0	99.0
**00020 Citrate cycle (TCA cycle)**	42	1089	0.7736618071841690	346.0	56150.0	142.0	62.0

**00030 Pentose phosphate pathway**	65	1189	0.5801059320016230	424.0	55899.0	425.0	194.0
**00040 Pentose and glucuronate interconversions**	69	1256	0.5896851476187490	457.0	55574.0	410.0	223.0
**00051 Fructose and mannose metabolism**	71	1506	0.5425171605375680	424.0	55891.0	370.0	328.0
**00052 Galactose metabolism**	64	1330	0.578369297572232	410.0	55245.0	381.0	214.0
**00053 Ascorbate and aldarate metabolism**	71	1325	0.5480372306228640	465.0	56062.0	517.0	251.0
**00061 Fatty acid biosynthesis**	23	670	0.5857307522988000	202.0	56524.0	270.0	48.0
**00062 Fatty acid elongation**	39	2179	0.6824945492484340	347.0	55567.0	339.0	27.0
**00071 Fatty acid degradation**	51	2484	0.6439211962872030	437.0	56211.0	423.0	92.0
**00073 Cutin, suberine and wax biosynthesis**	23	656	0.6142145231530840	185.0	56583.0	223.0	36.0
**00100 Steroid biosynthesis**	68	2069	0.8438381240718750	616.0	55703.0	131.0	94.0
**00120 Primary bile acid biosynthesis**	55	2299	0.8489302256003490	491.0	56404.0	124.0	51.0
**00121 Secondary bile acid biosynthesis**	36	1184	0.8496184733002620	345.0	56321.0	80.0	42.0
**00130 Ubiquinone and other terpenoid-quinone biosynthesis**	91	2339	0.7202567520407570	711.0	55463.0	313.0	228.0
**00140 Steroid hormone biosynthesis**	108	2745	0.8985746926606160	984.0	55486.0	163.0	57.0
**00190 Oxidative phosphorylation**	14	372	0.5561799510943540	81.0	56711.0	88.0	44.0
**00195 Photosynthesis**	6	184	0.5687909308210510	43.0	56722.0	49.0	19.0
**00220 Arginine biosynthesis**	29	605	0.7568809031535640	214.0	56472.0	49.0	89.0
**00230 Purine metabolism**	121	2755	0.8532967940978590	1212.0	55166.0	366.0	56.0
**00232 Caffeine metabolism**	28	563	0.8541798746855700	242.0	56951.0	26.0	57.0
**00240 Pyrimidine metabolism**	90	1787	0.8113729465430260	837.0	55378.0	293.0	97.0
**00250 Alanine, aspartate and glutamate metabolism**	51	1075	0.6216987663625870	310.0	56068.0	188.0	184.0
**00253 Tetracycline biosynthesis**	46	1468	0.7919846416467210	412.0	56190.0	192.0	34.0

**00254 Aflatoxin biosynthesis**	36	1093	0.9055626098952720	337.0	56389.0	44.0	26.0
**00260 Glycine, serine and threonine metabolism**	60	1074	0.632184821848565	397.0	56010.0	229.0	225.0
**00261 Monobactam biosynthesis**	49	1098	0.7165231174376000	353.0	56369.0	142.0	134.0
**00270 Cysteine and methionine metabolism**	96	1699	0.6882694148003660	721.0	55366.0	376.0	263.0
**00280 Valine, leucine and isoleucine degradation**	52	1633	0.6458800086230890	393.0	56501.0	311.0	127.0
**00290 Valine, leucine and isoleucine biosynthesis**	29	503	0.7687005955828100	241.0	56030.0	70.0	74.0
**00300 Lysine biosynthesis**	55	1231	0.675042799256142	391.0	55833.0	222.0	151.0
**00310 Lysine degradation**	70	1565	0.6013072452787590	428.0	55910.0	284.0	272.0
**00311 Penicillin and cephalosporin biosynthesis**	31	692	0.7169593911073640	222.0	56107.0	83.0	91.0
**00330 Arginine and proline metabolism**	77	1316	0.624971876536468	555.0	55507.0	468.0	202.0
**00331 Clavulanic acid biosynthesis**	17	319	0.5018821415057870	90.0	56570.0	69.0	111.0
**00332 Carbapenem biosynthesis**	45	1047	0.7562328936259140	348.0	56338.0	107.0	115.0
**00333 Prodigiosin biosynthesis**	29	784	0.7242515796302220	227.0	56171.0	98.0	74.0
**00340 Histidine metabolism**	61	1115	0.7109610395634370	449.0	55834.0	212.0	149.0
**00350 Tyrosine metabolism**	95	1600	0.7132958932262360	720.0	55563.0	365.0	206.0
**00360 Phenylalanine metabolism**	61	1463	0.5784794345723250	353.0	55826.0	288.0	219.0
**00361 Chlorocyclohexane and chlorobenzene degradation**	91	1206	0.784074944506343	769.0	55253.0	249.0	167.0
**00362 Benzoate degradation**	97	2614	0.6105447143364770	648.0	55191.0	490.0	319.0
**00363 Bisphenol degradation**	29	568	0.869213773716127	240.0	56730.0	43.0	29.0
**00364 Fluorobenzoate degradation**	40	817	0.7277840822453640	271.0	56572.0	81.0	121.0
**00365 Furfural degradation**	16	428	0.6696526147960390	113.0	56955.0	55.0	56.0

**00380 Tryptophan metabolism**	109	2126	0.7957896057720780	941.0	55135.0	262.0	209.0
**00400 Phenylalanine, tyrosine and tryptophan biosynthesis**	56	1015	0.6432877086056760	373.0	56476.0	183.0	225.0
**00401 Novobiocin biosynthesis**	37	1012	0.7470854287503790	298.0	56205.0	107.0	93.0
**00402 Benzoxazinoid biosynthesis**	16	291	0.7176017065480430	102.0	56445.0	40.0	40.0
**00403 Indole diterpene alkaloid biosynthesis**	38	1301	0.8302009318289570	325.0	56047.0	96.0	38.0
**00404 Staurosporine biosynthesis**	64	1779	0.8191649844663780	539.0	56090.0	133.0	102.0
**00405 Phenazine biosynthesis**	41	952	0.6381257912091950	280.0	56366.0	167.0	147.0
**00410 beta-Alanine metabolism**	45	973	0.7087123261934690	340.0	56524.0	158.0	119.0
**00430 Taurine and hypotaurine metabolism**	30	756	0.7607651217055730	237.0	56470.0	72.0	76.0
**00440 Phosphonate and phosphinate metabolism**	68	1453	0.7966198293069850	572.0	56491.0	187.0	103.0
**00450 Selenocompound metabolism**	31	655	0.6830059208336810	235.0	56240.0	151.0	70.0
**00460 Cyanoamino acid metabolism**	50	763	0.6116918933903410	325.0	56103.0	233.0	175.0
**00470 D-Amino acid metabolism**	79	1252	0.6598403086147240	563.0	55805.0	320.0	249.0
**00480 Glutathione metabolism**	53	1258	0.5799322025862940	308.0	56044.0	260.0	182.0
**00500 Starch and sucrose metabolism**	54	1346	0.6392912010311820	361.0	56104.0	249.0	156.0
**00510 N-Glycan biosynthesis**	26	1129	0.7291468418447760	200.0	56482.0	94.0	55.0
**00512 Mucin type O-glycan biosynthesis**	3	127	-0.00027401531865905300	0.0	56871.0	9.0	27.0
**00513 Various types of N-glycan biosynthesis**	4	73	0.027205888688917800	1.0	56796.0	37.0	33.0
**00514 Other types of O-glycan biosynthesis**	6	175	0.4525634034550170	24.0	56442.0	22.0	37.0
**00515 Mannose type O-glycan biosynthesis**	6	158	0.39863663629348100	25.0	56606.0	45.0	31.0

**00520 Amino sugar and nucleotide sugar metabolism**	137	4033	0.7401919779086580	1122.0	55133.0	547.0	230.0
**00521 Streptomycin biosynthesis**	39	969	0.7195865469126920	294.0	56349.0	121.0	106.0
**00522 Biosynthesis of 12-, 14- and 16-membered macrolides**	95	4281	0.8777338297470100	832.0	56092.0	137.0	91.0
**00523 Polyketide sugar unit biosynthesis**	73	2432	0.8319233194762350	672.0	55971.0	223.0	53.0
**00524 Neomycin, kanamycin and gentamicin biosynthesis**	103	3240	0.8811058059278500	892.0	55873.0	128.0	108.0
**00525 Acarbose and validamycin biosynthesis**	52	1224	0.8125023355285470	446.0	55946.0	132.0	73.0
**00531 Glycosaminoglycan degradation**	13	375	0.1117813630720040	13.0	56619.0	78.0	131.0
**00532 Glycosaminoglycan biosynthesis - chondroitin sulfate / dermatan sulfate**	4	114	0.6764558122534930	25.0	56932.0	10.0	14.0
**00534 Glycosaminoglycan biosynthesis - heparan sulfate / heparin**	2	58	0.49601170570954100	8.0	56530.0	18.0	2.0
**00540 Lipopolysaccharide biosynthesis**	71	5332	0.7119255155083730	579.0	55988.0	300.0	165.0
**00541 O-Antigen nucleotide sugar biosynthesis**	110	3764	0.8140831474534970	994.0	55373.0	376.0	84.0
**00550 Peptidoglycan biosynthesis**	41	2517	0.7096009771486460	337.0	56391.0	199.0	81.0
**00552 Teichoic acid biosynthesis**	40	2113	0.6129455314284880	292.0	56336.0	271.0	107.0
**00561 Glycerolipid metabolism**	50	1585	0.7288252640151510	411.0	55887.0	202.0	104.0
**00562 Inositol phosphate metabolism**	59	1456	0.7590277931409680	434.0	56088.0	130.0	142.0
**00563 Glycosylphosphatidylinositol (GPI)-anchor biosynthesis**	10	298	0.2623363639353410	36.0	56544.0	148.0	65.0
**00564 Glycerophospholipid metabolism**	76	1973	0.8329444010426630	686.0	55779.0	191.0	83.0
**00565 Ether lipid metabolism**	45	1002	0.6583909820237230	317.0	56322.0	245.0	92.0

**00571 Lipoarabinomannan (LAM) biosynthesis**	10	466	0.4353021170675020	55.0	56497.0	88.0	56.0
**00572 Arabinogalactan biosynthesis - Mycobacterium**	9	474	0.2429860220833520	28.0	56063.0	113.0	65.0
**00590 Arachidonic acid metabolism**	85	2186	0.9020556642167380	803.0	55749.0	144.0	31.0
**00591 Linoleic acid metabolism**	30	744	0.6694539415743330	251.0	56492.0	171.0	80.0
**00592 alpha-Linolenic acid metabolism**	62	2027	0.8213088985460710	529.0	55909.0	150.0	79.0
**00600 Sphingolipid metabolism**	56	1650	0.7081600596698010	490.0	55922.0	329.0	89.0
**00601 Glycosphingolipid biosynthesis - lacto and neolacto series**	26	2339	0.9355472092944740	255.0	56329.0	21.0	14.0
**00603 Glycosphingolipid biosynthesis - globo and isoglobo series**	11	673	0.8199876238303620	93.0	56348.0	26.0	15.0
**00604 Glycosphingolipid biosynthesis - ganglio series**	16	1368	0.8008036639157520	123.0	56686.0	29.0	32.0
**00620 Pyruvate metabolism**	51	1324	0.6833139881448820	377.0	55811.0	189.0	156.0
**00621 Dioxin degradation**	69	1294	0.8371759600896710	584.0	56071.0	102.0	122.0
**00622 Xylene degradation**	46	740	0.7119955738202290	335.0	56375.0	127.0	141.0
**00623 Toluene degradation**	56	1059	0.4611271845303230	272.0	55978.0	276.0	348.0
**00624 Polycyclic aromatic hydrocarbon degradation**	121	2254	0.7537280966208090	950.0	55356.0	340.0	264.0
**00625 Chloroalkane and chloroalkene degradation**	11	345	0.6468011531234290	75.0	56552.0	54.0	29.0
**00626 Naphthalene degradation**	69	1438	0.6357825704670690	404.0	55875.0	217.0	238.0
**00627 Aminobenzoate degradation**	106	2056	0.6457810046377090	721.0	55168.0	357.0	411.0
**00630 Glyoxylate and dicarboxylate metabolism**	78	1961	0.6352256427893340	553.0	55918.0	390.0	237.0
**00633 Nitrotoluene degradation**	33	658	0.8075168569911570	244.0	56672.0	20.0	101.0
**00640 Propanoate metabolism**	41	1216	0.5275516914956930	253.0	56251.0	284.0	169.0

**00642 Ethylbenzene degradation**	20	488	0.4856692068933750	70.0	56518.0	53.0	98.0
**00643 Styrene degradation**	27	628	0.5442834314614210	143.0	56398.0	90.0	151.0
**00650 Butanoate metabolism**	43	1339	0.6337107369408950	278.0	56370.0	185.0	134.0
**00660 C5-Branched dibasic acid metabolism**	40	872	0.5609187135182550	237.0	56121.0	225.0	145.0
**00670 One carbon pool by folate**	42	1048	0.5127876754159670	242.0	56678.0	305.0	159.0
**00680 Methane metabolism**	102	2534	0.7533470795904140	856.0	55473.0	424.0	140.0
**00710 Carbon fixation in photosynthetic organisms**	30	571	0.6911517217454610	239.0	56530.0	141.0	74.0
**00720 Carbon fixation pathways in prokaryotes**	48	1706	0.7372234377314740	403.0	56001.0	193.0	95.0
**00730 Thiamine metabolism**	34	598	0.6351616397377490	209.0	56495.0	94.0	146.0
**00740 Riboflavin metabolism**	36	928	0.6430606851335270	239.0	56313.0	120.0	143.0
**00750 Vitamin B6 metabolism**	42	664	0.68878962694997	301.0	56341.0	132.0	137.0
**00760 Nicotinate and nicotinamide metabolism**	77	1312	0.7566014310904930	609.0	55501.0	212.0	173.0
**00770 Pantothenate and CoA biosynthesis**	53	975	0.8125071653405430	477.0	55856.0	163.0	59.0
**00780 Biotin metabolism**	29	704	0.6510390615787090	218.0	56140.0	173.0	67.0
**00785 Lipoic acid metabolism**	41	1160	0.6104343767640310	333.0	56235.0	337.0	106.0
**00790 Folate biosynthesis**	81	1941	0.7820022413017530	663.0	55408.0	244.0	123.0
**00791 Atrazine degradation**	22	267	0.8879918741580400	225.0	56344.0	40.0	17.0
**00830 Retinol metabolism**	33	1005	0.6565859869886410	275.0	56211.0	271.0	44.0
**00860 Porphyrin metabolism**	161	7551	0.7786835720932580	1439.0	54827.0	577.0	225.0
**00900 Terpenoid backbone biosynthesis**	58	1676	0.6983224177776770	470.0	55767.0	285.0	124.0
**00901 Indole alkaloid biosynthesis**	96	2470	0.8787511753723070	887.0	55879.0	154.0	87.0
**00902 Monoterpenoid biosynthesis**	70	1166	0.5985305822686890	412.0	55937.0	285.0	257.0

**00903 Limonene degradation**	56	1156	0.8168080310251450	433.0	55751.0	97.0	95.0
**00904 Diterpenoid biosynthesis**	135	3445	0.8595465627286350	1181.0	55543.0	237.0	140.0
**00905 Brassinosteroid biosynthesis**	33	1088	0.9358212201321070	307.0	56710.0	28.0	14.0
**00906 Carotenoid biosynthesis**	142	5553	0.8970744191367720	1348.0	55336.0	201.0	101.0
**00907 Pinene, camphor and geraniol degradation**	65	2056	0.7458763981009230	497.0	56128.0	200.0	135.0
**00908 Zeatin biosynthesis**	38	964	0.7921510901356950	312.0	56427.0	98.0	65.0
**00909 Sesquiterpenoid and triterpenoid biosynthesis**	92	2033	0.6272931720065450	635.0	55392.0	417.0	319.0
**00910 Nitrogen metabolism**	15	453	0.6766126048746800	96.0	56451.0	55.0	37.0
**00920 Sulfur metabolism**	52	1345	0.6747486742538950	332.0	55763.0	162.0	154.0
**00930 Caprolactam degradation**	35	827	0.5605678597768910	201.0	56555.0	186.0	128.0
**00940 Phenylpropanoid biosynthesis**	74	1812	0.7649315369779430	618.0	55988.0	238.0	138.0
**00941 Flavonoid biosynthesis**	92	2361	0.7736911928778960	761.0	55662.0	287.0	153.0
**00942 Anthocyanin biosynthesis**	79	3666	0.8521201133396060	702.0	55893.0	155.0	86.0
**00943 Isoflavonoid biosynthesis**	76	1902	0.8394004082119710	665.0	56336.0	149.0	102.0
**00944 Flavone and flavonol biosynthesis**	66	2332	0.8267688659404890	637.0	56071.0	210.0	60.0
**00945 Stilbenoid, diarylheptanoid and gingerol biosynthesis**	34	1108	0.75979790751362	263.0	56251.0	110.0	57.0
**00946 Degradation of flavonoids**	47	1048	0.48029316462370500	239.0	56494.0	286.0	224.0
**00950 Isoquinoline alkaloid biosynthesis**	144	3669	0.9095550637821420	1371.0	55811.0	148.0	117.0
**00960 Tropane, piperidine and pyridine alkaloid biosynthesis**	91	1891	0.793513754160815	730.0	55647.0	213.0	160.0
**00965 Betalain biosynthesis**	32	895	0.70684621101882	215.0	56478.0	87.0	90.0

**00966 Glucosinolate biosynthesis**	89	1622	0.8882234493070090	798.0	55783.0	149.0	51.0
**00980 Metabolism of xenobiotics by cytochrome P450**	115	2346	0.8206921470458940	897.0	55710.0	180.0	203.0
**00981 Insect hormone biosynthesis**	33	950	0.770038639507383	309.0	55970.0	144.0	45.0
**00982 Drug metabolism - cytochrome P450**	94	1809	0.9115830208550640	845.0	55678.0	84.0	77.0
**00983 Drug metabolism - other enzymes**	74	1677	0.832535891433835	590.0	56134.0	106.0	128.0
**00984 Steroid degradation**	29	811	0.780509722686123	245.0	56261.0	76.0	61.0
**00996 Biosynthesis of various alkaloids**	89	2537	0.6769750135188250	632.0	55466.0	351.0	241.0
**00997 Biosynthesis of various other secondary metabolites**	82	1720	0.620256681819166	581.0	55712.0	467.0	240.0
**00998 Biosynthesis of various antibiotics**	126	2586	0.7469649931826780	960.0	55172.0	320.0	311.0
**00999 Biosynthesis of various plant secondary metabolites**	174	5573	0.7524500635441920	1378.0	54640.0	512.0	360.0
**01040 Biosynthesis of unsaturated fatty acids**	80	4509	0.7931406916340190	765.0	55455.0	406.0	23.0
**01051 Biosynthesis of ansamycins**	48	1614	0.7428984261088510	389.0	56201.0	157.0	110.0
**01052 Type I polyketide structures**	25	1280	0.42387899618925300	87.0	56704.0	84.0	157.0
**01053 Biosynthesis of siderophore group nonribosomal peptides**	29	809	0.6271569045656970	183.0	56929.0	101.0	115.0
**01054 Nonribosomal peptide structures**	14	1062	0.6934693151337760	72.0	56579.0	6.0	66.0
**01055 Biosynthesis of vancomycin group antibiotics**	26	909	0.4828061733893560	129.0	56358.0	156.0	119.0
**01056 Biosynthesis of type II polyketide backbone**	30	1034	0.8639344763724760	268.0	56292.0	48.0	36.0
**01057 Biosynthesis of type II polyketide products**	160	5716	0.9271790666316450	1429.0	55208.0	119.0	99.0
**01059 Biosynthesis of enediyne antibiotics**	73	2401	0.7364613478070240	546.0	55934.0	200.0	184.0
**01060 Biosynthesis of plant secondary metabolites**	134	2511	0.576574875334981	998.0	53880.0	1125.0	355.0

**01061 Biosynthesis of phenylpropanoids**	105	2123	0.5836969274582290	779.0	55420.0	822.0	299.0
**01062 Biosynthesis of terpenoids and steroids**	96	2135	0.5477297998792720	641.0	55026.0	727.0	328.0
**01063 Biosynthesis of alkaloids derived from shikimate pathway**	142	2962	0.7421287384147680	1150.0	54903.0	546.0	240.0
**01064 Biosynthesis of alkaloids derived from ornithine, lysine and nicotinic acid**	63	993	0.7425623581569070	499.0	55896.0	220.0	124.0
**01065 Biosynthesis of alkaloids derived from histidine and purine**	34	565	0.7307892738408580	280.0	56389.0	141.0	67.0
**01066 Biosynthesis of alkaloids derived from terpenoid and polyketide**	47	1161	0.5083709282611360	226.0	56450.0	201.0	230.0
**01070 Biosynthesis of plant hormones**	66	1454	0.46495064652427200	396.0	55407.0	724.0	231.0
